# Early sexual debut and associated factors among secondary school students of central zone of Tigray, Northern Ethiopia, 2018

**DOI:** 10.11604/pamj.2019.34.1.17139

**Published:** 2019-09-01

**Authors:** Alem Girmay, Teklewoini Mariye, Hadgu Gerensea

**Affiliations:** 1 Department of Adult health Nursing, School of Nursing, College of Health Science and Comprehensive Specialized Hospital, Aksum University, Tigray, Ethiopia; 2 Department of Pediatric and Child Health Nursing, School of Nursing, College of Health Science and Comprehensive Specialized Hospital, Aksum University, Tigray, Ethiopia

**Keywords:** Sexual debut, adolescent, Ethiopia

## Abstract

**Introduction:**

Early sexual debut is common among young people and it has several sexual and reproductive health consequences. But, its burden and the associated factors leading to this behavior haven't received due attention. The main purpose of this study was to investigate the prevalence and associated factors of sexual debut in the preparatory and high school students of Aksum town.

**Methods:**

A school-based quantitative cross-sectional study design was used for this research work. A total of 519 preparatory and high school regular students participated in the survey. The sample population was obtained by using a simple random sampling technique from each schooling proportion with their number of students. Data, that were collected using self administered questionnaires, were entered into EpiData 3.02 and analyzed in SPSS 22.0. Results were presented using frequencies, tables and graphs. Statistical significance was declared at a P-value <0.05.

**Results:**

Of the total participants, 266 (51.3%) were males. The age of the participants ranged from 13 to 23 years with a mean age of 16.3 ± 1.47 years. Of the total participants, 137(26.2%) had sexual experience, among which 119 (87.5%) had an early sexual debut at an average age of 13.7 + 1.4 years. Factors that were found to be significantly associated with an early sexual debut were gender (AOR=3.41; 95% CI: 1.54, 6.99), residence (AOR=0.44; 95% CI: 0.27, 0.81), alcohol drinking (AOR=5.5; 95% CI: 2.2, 14.8), cigarette smoking (AOR=3.3; 95% CI: 2.3, 7.5), exposure to pornography, such as reading/seeing pornographic materials (AOR=7.4; 95% CI: 4.4, 11.78), living arrangement for educational purpose (AOR= 0.43; 95% CI: 0.13, 0.89), grade (AOR=0.38; 95% CI: 0.06, 0.68) and monthly living allowance (AOR=0.419; 95% CI: 0.2, 0.9).

**Conclusion:**

A significant number of students reported early sexual debut. Gender, place of residence, alcohol drinking, cigarette smoking, exposure to pornography, grade and living arrangement for educational purpose and monthly living allowance were significant predictors of an early sexual debut.

## Introduction

Adolescence is a period of human development that everyone passes through and experiences new situations as well as experiments different life events. The result of these experiments might be good or bad depending on the different personal and environmental factors [1]. Almost half of the world's population's age is concentrated under the age of 25 years. Most of this segment of the population lives in developing countries with Sub-Saharan Africa youths constituting 20%-30% of this population. According to the 2007 Ethiopian census, youths aged 15-24 years contribute to 20.6% of the whole population (more than 15.2 million). Early sexual debut is common among young people and it has several sexual and reproductive health consequences such as sexual coercion, female genital cutting, unplanned pregnancies, closely spaced pregnancies, abortion, sexually transmitted infections (STIs) and HIV/AIDS. Each of these consequences contributes to further other physical, psychological, social and economic problems [2-5].

Around 11% of the worldwide births are from people less than 20 years of age. Of this, 95% were reported from developing countries with Ethiopia being one of the countries with a high birth rate from this age group. Internationally, around 26 million adolescent pregnancies occur yearly and abortion, miscarriage or still birth happened in 10 million of those. Findings from demographic health surveys from developing countries indicated that about 10% of adolescent girls became mothers at 16 years old, the majority of them being from sub-Saharan Africa and South-Central and South-Eastern Asia [6]. Adolescents, which constitutes are sizable proportion worldwide, are exposed to many health problems. For instance, around 60,000 adolescent women die every year related to these health problems. Worldwide, around one million pregnancies happen each day; of this, 50% are out of plan and 25% are unwanted pregnancies [7]. Different studies were done in different regions of the world to identify the prevalence and associated factors of early sexual debut. Based on the finding from these studies, sexual activities among youths and adolescents have been reported to be increasing worldwide [5]. Despite the significance of the worldwide magnitude of early sexual debut, no research had been done to identify the prevalence and associated factors of early sexual debut in the study area of this research. It is anticipated that investigating this behavior and associated factors in this study area will contribute to identifying the magnitude and factors which may be utilized by schools, zonal and regional health bureaus to design appropriate interventions based on the finding.

## Methods

**Study area and design:** school-based cross-sectional study was conducted from March 1 to March 30, 2018, in the Central Zone of Tigray, Northern Ethiopia in public preparatory and high schools. This is located 1024 km away from the capital city of Addis Ababa. There are 2 preparatory and 3 secondary public schools found in the town, with the total students of 6,939 during the study period.

**Sample size:** single population proportion was used to calculate the sample size in Epi Info version 7 with the following assumptions: prevalence= 19% [8], 95% CI, a margin of error 5% for a prevalence of early sexual debut and power=80% and the ratio of unexposed to exposed was almost =1. A sample of 519 students (initially 236 samples, 2 design effect and 10% non-response rate) was taken randomly after stratification was made according to their grade level.

**Data collection tool and analysis:** data were collected using semi-structured self-administered questionnaire. This included the respondents' Socio-demographic characteristics, parent characteristics as well as other pertinent data such as alcohol drinking, cigarette smoking and living arrangement for educational purpose, monthly living allowance and exposure to pornography. Training was given for data collectors about the purpose and procedures of the study. To control the quality of the data, double data entry was done by two data clerks and the consistency of the entered data was cross-checked by comparing the two separately entered data on Epi Data. The raw data was cleaned, coded and entered into the computer as soon as data were generated and analyzed using SPSS version 22. Data were summarized using descriptive and inferential statistics. Bivariate analysis at 95% confidence interval was used to infer an association between the independent and outcome variables. Independent factors, with a P-value ≤ 0.25 in the bivariate logistic regression, were entered into the multiple logistic regression model to obtain the significant variables. Statistical significance was declared at P< 0.05 and variables which were statistically significant at p-value < 0.05 were identified as factors of early sexual debut.

### Operational definition

**Early sexual debut:** having sexual intercourse at or before the age of 14 years old [9].

**Ethical approval and consent to participate:** this study was reviewed and approved by the Research Committee, Health Science College and Comprehensive Specialized Hospital of Aksum University. Consent was also obtained from both the educational bureau of the town and directors of each school. The objective and importance of the study were explained clearly to the study participants. Moreover, participants were informed that their participation is not compulsory and can leave the participation at any time during the interview. Respondents were also assured that their information will be kept confidential. Data were collected after full informed oral consent/assent was obtained from each study subjects whose age was less than 16 years. In order to keep the confidentiality of respondents' data, their name was kept anonymous.

## Results

**Socio-demographic characteristics of participants**: a total of 519 students participated in the study with 100% response rate. From the participants 266 (51.3%) were males and 253 (48.7%) were females. Their age ranged from 13 to 23 years with a mean age of 16.3 and SD of ± 1.47 years (16.62 ± 1.7 and 16.12 ± 1.2 for males and females respectively). The majority of the students, 330(63.6%), were in the age group of 15-17 years and were Tigrayan (461, 88.8%) in ethnicity. 228 of them (43.9%) were from grade ten and many of the students, 409 (78.8%), were urban residents. The majority of the students (473, 91.1%) were Orthodox religion followers. Around 240 (46.2%) fathers and 217(41.8%) mothers of the students can read and write. Most of the students, 458 (88.2%), were living with their family. 367 (70.7%) students were getting less than or equal to 5.5 US dollars as the monthly living allowance. 120 (23.1%) and 23(4.4%) of the students drink alcohol and smoke cigarettes, respectively. 168 (32.4%) students had seen/read pornographic materials and 29(5.6%) had experienced having sex with commercial sex workers (Table 1).

**Table 1:** Participants characteristics in the study on early sexual debut and associated factors among secondary school students of Aksum town, Central Zone of Tigray, Northern Ethiopia, 2018(n=519)

Variables	Frequency (%)
**Age in year**	
<15	158(30.4)
15-17	330(63.6)
>=18	30(5.8)
**Gender**	
Male	253(48.7)
Female	266(51.3)
**Ethnicity**	
Tigray	461(88.8)
Amhara	39(7.5)
Oromo	19(3.7)
**Grade Level**	
Grade 9	203(39.1)
Grade 10	228(43.9)
Grade 11	55(10.6)
Grade 12	33(6.4)
**Place of Residence**	
Urban	409(78.8)
Rural	110(21.2)
**Religion**	
Orthodox	473(91.1)
Muslim	33(6.4)
Protestant	12(2.3)
Others	1(0.2)
**Educational level of father**	
Can’t read and write	41(7.9)
Read and write	240(46.2)
Elementary	145(29.7)
High school, College and above	92(17.7)
**Educational level of mother**	
Can’t read and write	108(20.8)
Read and write	217(41.8)
Elementary	125(24.1)
High school College and above	68(13.1)
**Fathers occupation**	
Employed	256(49.3)
Unemployed	259(49.9)
**Mothers occupation**	
Employed	172(33.1)
Unemployed	345(66.5)

**Prevalence of early sexual debut:** of the total 519 participants, 136 (26.2%) had experienced sex in their life. Among them, 119 (87.5%) had an early sexual debut with average sexual debut age of 13.7 + 1.4 (Table 1).

**Factors associated with early sexual debut:** independent variables such as gender, place of residence, exposure to pornography, father's educational level, mother's educational level, alcohol drinking, cigarette smoking, living arrangement for educational purpose, monthly living allowance and the students' grade were associated with early sexual debut at p-value < 0.25 in the binary logistic regression. To get the final determinants of the outcome variable, the above variables were entered into multivariate logistic regression analysis and finally, sex, residence, alcohol drinking, cigarette smoking, exposure to pornography, living arrangement for educational purpose, grade and monthly living allowance had a significant association with an early sexual debut of students (Table 2).

**Table 2:** Factors associated with early sexual debut among secondary school students of Aksum town, Central Zone of Tigray, Northern Ethiopia, 2018 (n=519)

Variables	Category	Early sexual debut		p-value 95%CI COR		p-value 95% CI AOR	
		Yes	No				
Place of Residence**	Rural	15	322		1		1
	Urban	104	78	0.199	0.035(0.24, 0.98)	0.004	0.44 (0.27, 0.81)
Gender**	Male	64	189	0.004	1.3(1.2, 2.7)	0.023	3.41 (1.54, 6.99)
	Female	55	211		1		1
Exposure to Pornography**	Yes	76	92	0.001	6(3.9, 9.2)	0.001	7.4 (4.4, 11.78)
	No	43	307		1		
**Fathers education**	Can’t read and write	13	28	0.135	3.4(1.2, 4.1)	0.115	2.6(0.79, 8.3)
	Read and write	65	192	0.961	2.5(0.6, 1.7)	0.518	1.3(0.55, 3.2)
	Elementary	51	94	0.459	3.9(0.7, 2.2)	0.322	1.6(0.64, 3.79)
	High school and above	11	81		1		1
**Mothers education**	Can’t read and write	11	16	0.501	1.29(0.6, 2.8)	0.794	0.9(0.28, 2.6)
	Read and write	39	15	0.425	1.3(0.7,2.5)	0.530	1.4(0.5, 3.8)
	Elementary	29	10	0.094	1.8(1.3, 2.6)	0.388	1.6(0.56, 4.3)
	High school and above	10	4		1		1
Alcohol drinking**	Yes	51	69	0.001	2.7(1.7, 4.2)	0.001	5.5 (2.2, 14.8)
	No	85	314		1		1
Cigarette smoking**	Yes	16	7	0.001	1.41(1.05, 3.32)	0.04	3.3 (2.3, 7.5)
	No	120	376	1	1		1
Pocket money**	<5.5 US dollar	59	19	0.003	0.4(0.23, 0.74)	0.016	0.419(0.2, 0.9)
	5.6-9.25 US dollar	10	12	0.39	0.72(0.34, 1,5)	0.259	0.39(0.25, 1.4)
	9.3-18.5 US dollar	3	9	0.88	0.92(0.38, 2.2)	0.553	1.5(0.48, 3.97)
	>18.5 US dollar	12	10	1	1		1
Living arrangement for education purpose**	Family	340	118	0.33	2.8(0.34, 22.4)	0.034	0.43(0.13, 0.89)
	Other relatives	15	9	0.169	4.8(1.5,44.9)	0.001	4.2(2.4, 6.3)
	Rental/my effort	28	9	1	1		1
Grade**	Grade 9	162	41	0.001	0.24(0.11, 0.51)	0.0001	0.38(0.06, 0.68)
	Grade 10	169	59	0.03	0.39(0.16, 0.69)	0.0003	0.14(0.08, 48)
	Grade 11	36	19	0.119	0.497(0.2, 1.2)	0.019	0.16(0.1, 0.8)
	Grade 12	16	17	1	1		1

## Discussion

This study investigated the magnitude of early sexual debut and its determinants among preparatory and high school regular students of Aksum town. The prevalence of having sex in their life in the study area was 26.2%. This finding was higher than the finding from Northern Ethiopia [7] and Nigeria [10] but lower than the studies in South West Ethiopia [11], United States [12], Tanzania [13], Kenya [14], Debremarkos, Ethiopia [15] and EDHS, 2016 [16]. This may be because of the sample size differences, the study area and the study population. Among the students who had experienced sex, 87.5% had an early sexual debut. This finding was higher than the finding in Tanzania, 57.8% [13] and South Africa, 39% [17]. In this study, the mean age of an early sexual debutant was 13.7 + 1.4. This finding was lower than the results from a study done in Debremarkos, Ethiopia and higher than the finding from Nigeria, South Africa and Tanzania [10, 13, 15, 17]. This study also showed that the mean age of early sexual debut was lower among females (13.4 + 1.5) than their male counterparts (14 + 0.5). This was in line with the study done in Nigeria [10].

Factors associated with early sexual debut: as revealed in the result, place of residence was significantly associated with early sexual debut of students. Respondents from the urban residence were 0.44 times less likely to be an early sexual debutante than those from the rural areas (AOR=0.44; 95% CI: 0.27, 0.81). This is in line with a study done in Debremarkos, Ethiopia [15]. This study found that gender was significantly associated with early sexual debut. Male students were 3.4 times more likely to be an early sexual debutante than female students (AOR=3.41; 95% CI: 1.54, 6.99). This is in line with a study conducted in Northern Ethiopia, Nigeria and South Africa and in contrast to the study result in Debremarkos, Ethiopia and in Thailand [7, 10, 15, 17, 18]. Exposure to pornography, such as reading/seeing pornographic materials, was significantly associated with an early sexual debut. Respondents who were exposed to pornography were 7.4 times more likely to be an early sexual debutante than those that had no exposure to pornography (AOR=7.4; 95% CI: 4.4, 11.78). This is comparable to findings from Debremarkos, Ethiopia, Bahr dar, Ethiopia, North-East Ethiopia [4, 19, 20]. Alcohol drinking was strongly associated with an early sexual debut of high school and preparatory school students. Those who drink alcohol were 5.5 times more likely to be an early sexual debutante than the non-alcohol drinkers, (AOR=5.5; 95% CI: 2.2, 14.8). This is in line with the study in Northern Ethiopia, Kenya, Thailand, Nigeria, Tanzania, Debremarkos Ethiopia, South East Ethiopia and North-East Ethiopia [1, 7, 10, 13-15, 18, 20].

Cigarette smoking among the students was significantly associated with an early sexual debut. Students who smoke cigarette were 3.3 times more likely to be an early sexual debutante than non- smokers, (AOR=3.3; 95% CI: 2.3, 7.5). This was comparable to the study conducted in Kenya, Thailand and North East Ethiopia [14, 18, 20]. Living with family had a significant association with an early sexual debut. Students who live with their families were 0.43 times less likely to be an early sexual debutante than those who were not living with their family, (AOR= 0.43; 95% CI: 0.13, 0.89). This was in line with the study conducted in Eastern Ethiopia [21]. The students' monthly living allowance was significantly associated with early sexual debut. Students who had ≤ 5.5 US dollar pocket money per month were 0.42 times less likely to be an early sexual debutante than those who had greater than 18.5 US dollars, (AOR=0.419; 95% CI: 0.2, 0.9). This is comparable with a study done in northern Ethiopia [7]. The grade of the students was significantly associated with an early sexual début. Students in the lower grade (grade 9), were 0.4 times less likely to be an early sexual debutante than those in the higher grade level, (AOR=0.38; 95% CI: 0.06, 0.68). This may be due to the fact that lower grade students are usually younger in age and hence have less knowledge about sex than students in the higher grade. This means that being in the lower grade decreases early sexual debut by 17%.

## Conclusion

A significant number of students had an early sexual debut, the magnitude being 88 out of 100 students. Gender, place of residence, alcohol drinking, cigarette smoking, exposure to pornography, living arrangement for educational purpose, grade and monthly living allowance had a significant association with an early sexual debut of students.

**Limitation of the study:** since this study is a cross-sectional study, it does not show the direction of relationships among variables and because the subject is quite a sensitive one, some significant information might be under-reported.

### What is known about this topic

- Prevalence of early sexual debut among preparatory and high school students in Africa is high and it is a major public health problem;
- It has become the commonest cause of sexually transmitted infections, especially HIV/AIDS and other reproductive health problems on the continent, especially in Ethiopia;
- High school students are not getting due attention to their reproductive health and if nothing is done about it, many problems will happen in the future of these population groups.

### What this study adds

- Gender, place of residence, alcohol drinking, cigarette smoking, exposure to pornography and living arrangement for educational purpose, grade level and monthly living allowance were independent variables that were significantly associated with an early sexual debut of high school students;
- It is necessary that policy-makers and health care personnels focus on high school students' awareness creation about reproductive health problems and their possible solutions;
- Another research should be carried out to determine the prevalence of early sexual debut and to identify factors associated with it among high school students in a broader social context and larger sample size.

## Competing interests

This manuscript maintains no competing financial interest declaration from any person or Organization or non-financial competing interests such as political, personal, religious, ideological, academic, intellectual, commercial or any other.
